# *MicroRNA-148a* induces apoptosis and prevents angiogenesis with bevacizumab in colon cancer through direct inhibition of *ROCK1*/*c-Met* via *HIF-1α* under hypoxia

**DOI:** 10.18632/aging.204243

**Published:** 2022-08-22

**Authors:** Hsiang-Lin Tsai, Yueh-Chiao Tsai, Yen-Cheng Chen, Ching-Wen Huang, Po-Jung Chen, Ching-Chun Li, Wei-Chih Su, Tsung-Kun Chang, Yung-Sung Yeh, Tzu-Chieh Yin, Jaw-Yuan Wang

**Affiliations:** 1 Division of Colorectal Surgery, Department of Surgery, Kaohsiung Medical University Hospital, Kaohsiung Medical University, Kaohsiung 80708, Taiwan; 2 Department of Surgery, Faculty of Medicine, College of Medicine, Kaohsiung Medical University, Kaohsiung 80708, Taiwan; 3 Graduate Institute of Clinical Medicine, College of Medicine, Kaohsiung Medical University, Kaohsiung 80708, Taiwan; 4 Division of Trauma and Surgical Critical Care, Department of Surgery, Kaohsiung Medical University Hospital, Kaohsiung Medical University, Kaohsiung 80708, Taiwan; 5 Department of Emergency Medicine, Faculty of Post-Baccalaureate Medicine, College of Medicine, Kaohsiung Medical University, Kaohsiung 80708, Taiwan; 6 Graduate Institute of Injury Prevention and Control, College of Public Health, Taipei Medical University, Taipei 11031, Taiwan; 7 Division of General and Digestive Surgery, Department of Surgery, Kaohsiung Medical University Hospital, Kaohsiung Medical University, Kaohsiung 80708, Taiwan; 8 Department of Surgery, Kaohsiung Municipal Tatung Hospital, Kaohsiung Medical University, Kaohsiung 80145, Taiwan; 9 Graduate Institute of Medicine, College of Medicine, Kaohsiung Medical University, Kaohsiung 80708, Taiwan; 10 Center for Cancer Research, Kaohsiung Medical University, Kaohsiung 80708, Taiwan; 11 Pingtung Hospital, Ministry of Health and Welfare, Pingtung 90054, Taiwan

**Keywords:** apoptosis, anti-angiogenesis, miR-148a, bevacizumab, ROCK1/c-Met

## Abstract

Angiogenesis and antiapoptosis effects are the major factors influencing malignancy progression. Hypoxia induces multiple mechanisms involving microRNA (miRNA) activity. Vascular endothelial growth factor (VEGF) is correlated with angiogenesis. An antiapoptotic factor, myeloid leukemia 1 (Mcl-1) is the main regulator of cell death. This study examined the role of *miR-148a* in inhibiting VEGF and Mcl-1 secretion by directly targeting *ROCK1/c-Met* by downregulating *HIF-1α* under hypoxia. The protein expression of ROCK1 or Met/HIF-1α/Mcl-1 in HCT116 and HT29 cells (all *P* < 0.05) was significantly reduced by *miR-148a*. The tube-formation assay revealed that *miR-148a* significantly suppressed angiogenesis and synergistically enhanced the effects of bevacizumab (both *P* < 0.05). The MTT assay revealed the inhibitory ability of *miR-148a* in HCT116 and HT29 cells (both *P* < 0.05). *miR-148a* and bevacizumab exerted synergistic antitumorigenic effects (*P* < 0.05) in an animal model. Serum *miR-148a* expression of metastatic colorectal cancer (mCRC) patients with a partial response was higher than that of mCRC patients with disease progression (*P* = 0.026). This result revealed that *miR-148a* downregulated *HIF-1α/VEGF* and *Mcl-1* by directly targeting *ROCK1/c-Met* to decrease angiogenesis and increase the apoptosis of colon cancer cells. Furthermore, serum *miR-148a* levels have prognostic/predictive value in patients with mCRC receiving bevacizumab.

## INTRODUCTION

The third most common type of gastrointestinal cancer is colorectal cancer (CRC). It is also the third leading cause of cancer-induced deaths worldwide. It affects more than 900,000 patients each year [1–3]. The more progressive screening techniques and treatment modalities were used to decline the mortality rate of CRC. The metastatic colorectal cancer (mCRC) patients are used with various combinations of chemotherapeutic drugs and biologics, including vascular endothelial growth factors inhibitors (VEGF inhibitors; e.g., bevacizumab) and epidermal growth factor receptors inhibitors (EGFR inhibitors; e.g., cetuximab) [4]. The current standard treatment improves outcomes in most patients with mCRC but fails to provide prominent benefits in a notable proportion of individuals who experience drug-associated toxicities. Thus, it is imperative to develop predictive biomarkers that can help select patients who might benefit from such treatment. Accordingly, patients who might not obtain any benefit from such treatment can avoid drug-related toxicity and receive alternative treatment [5].

Angiogenesis is a complex process and a critical pace in the progression of malignancies. One of the symbols of cancer progression is angiogenesis [6, 7]. Apoptosis is a critical homeostatic mechanism that helps keep cell populations during normal cell development. Disruption in apoptosis regulation might lead to cancer [8]. MicroRNAs (miRNAs), noncoding RNAs, mediate the genetic expressions at the posttranscriptional level and play essential roles in tumorigenesis, cancer progression, and clinical therapy in various malignancies, one of which is CRC [2, 9, 10]. In addition, miRNAs, such as oncogenes and tumor suppressor genes, contribute to the proliferation, apoptosis, angiogenesis, invasion, and tumor metastasis of various cancers [11–13]. Hypoxia has been implicated in the pathogenesis of CRC and other malignancies. It can prevent apoptosis and result in cancer growth, anti-apoptosis, recurrence, and poor survival [14]. Li and his colleagues suggested in a review that *miR-148a* inhibits the proliferation, apoptosis, metastasis, and invasion of cancer cells by directly targeting *ROCK1* and *BCL-2* [15, 16]. A study demonstrated that *miR-148a* suppresses the epithelial-mesenchymal transition (EMT) of hepatocellular carcinoma by targeting *c-Met* [17]. We have previously observed that *miR-148a* inhibits tumorigenesis and reduces the likelihood of early CRC recurrence [18], improves the response to chemoradiation and increases apoptosis by directly targeting c-Met in patients with rectal cancer [19], indirectly inhibits VEGF secretion by targeting *HIF-1α* [7], and exhibits the apoptotic effect of alterations of myeloid cell leukemia 1 (Mcl-1) expression on CRC [20].

Xu et al. demonstrated in 2018 that chemokine CC ligand 19 (CCL19) inhibits CRC angiogenesis by promoting *miR-206* and thereby inhibiting the Met/ERK/HIF-1α/VEGF-A pathway [21]. Gluck et al. proved that Met regulates HIF-1α levels via a protein translation mechanism [22]. A previous study found that the Rho/ROCK pathway is essential to HIF-1α expression in ovarian cancer cell lines and is an upstream regulator of HIF-1α accumulation in ovarian cancer [23]. In 2020, Wu et al. reported that HIF-1α could promote Mcl-1 expression to act as a transcription factor by directly target the promoter region of Mcl-1 [24].

In this current study, we supposed that *miR-148a* directly targets *ROCK1* and *c-Met* to decrease angiogenesis and increase the apoptosis of colon cancer cells by inhibiting the secretion of VEGF and Mcl-1 through downregulation of *HIF-1α* under hypoxia. We also searched out the potential synergistic effects of the combination of *miR-148a* and bevacizumab.

## RESULTS

### *miR-148a* posttranscriptional directly reduced *ROCK1* and *c-Met* expression by targeting its 3′-UTR

We explored the mechanisms underlying the antimetastatic functions of *miR-148a*. The predicted target genes of *miR-148a* were gained from the TargetScan database (https://targetscan.org) and the isomiRTar portal (https://isomirtar.hse.ru/), especially those that can suppress angiogenesis and apoptosis in cancer cells. Thus, two candidate genes, *ROCK1* and *c-Met*, were selected (Supplementary Figure 1A, 1B).

**Figure 1:**
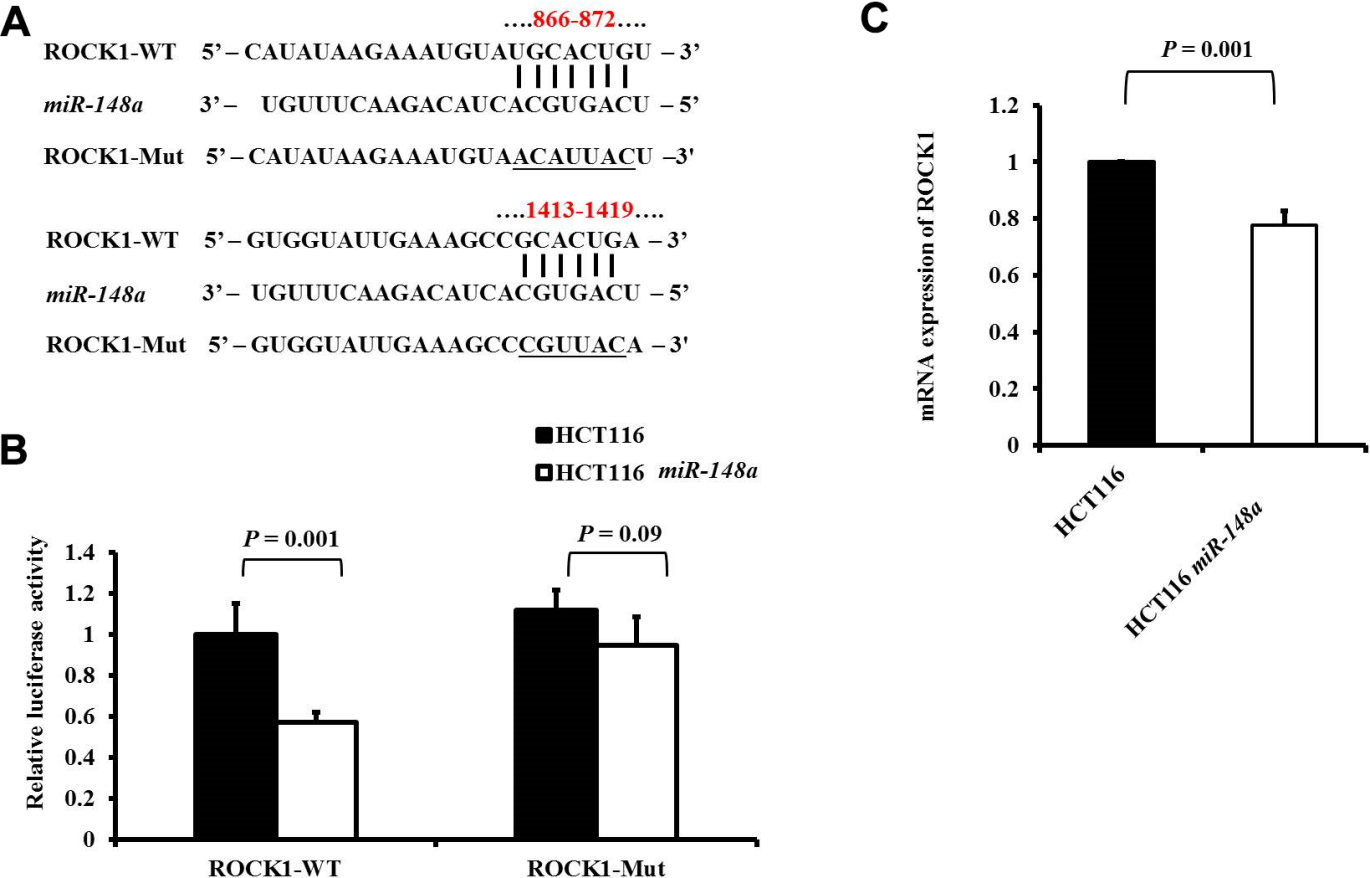
***ROCK1* is a direct target of *miR-148a* in HCT-116 cells.** (**A**) Two putative *miR-148a*-binding sites in *ROCK1* 3′-UTR and the two corresponding mutant binding sites (underlined) are shown; (**B**) *miR-148a* overexpression suppressed the activity of firefly luciferase that carried the wild-type (*P* = 0.001) but not mutant 3′-UTR of *ROCK1*; (**C**) The mRNA levels of *ROCK1* were determined using qRT-PCR in stable HCT116 and HCT116-*miR-148a* cell lines, and the difference was significant in the HCT116-*miR-148a* cell line (*P* = 0.001).

ROCK1, an effector kinase of Rho GTPases, plays a vital role in the regulation of cancer invasion and metastasis [25, 26]. Two possible binding sites on the 3′-UTR sequence of *ROCK1* for *miR-148a* were identified using TargetScan (https://targetscan.org). A series of 3′-UTR fragments of *ROCK1* including the full sequence, binding site1, binding site2, and their corresponding mutant counterparts were directly fused downstream of the firefly luciferase gene to assess whether *ROCK1* is a direct target of *miR-148a.* We co-transfected the mutant luciferase 3′-UTR construct into HCT116 and HCT116-*miR-148a* cells (Figure 1A). The luciferase activity of *ROCK1* was significantly decreased in the HCT116-*miR-148a* cell line transfected with wild-type *ROCK1* 3′-UTR (*P* = 0.001) but not in that transfected with mutant *ROCK1* 3′-UTR (*P* = 0.09; Figure 1B). A comparison of the mRNA expression of *ROCK1* between the HCT116 cells and HCT116-*miR-148a* cells revealed that *miR-148a* remarkably downregulated the expression of *ROCK1* mRNA in the HCT116-*miR-148a* cells (*P* = 0.001; Figure 1C).

**Figure 2:**
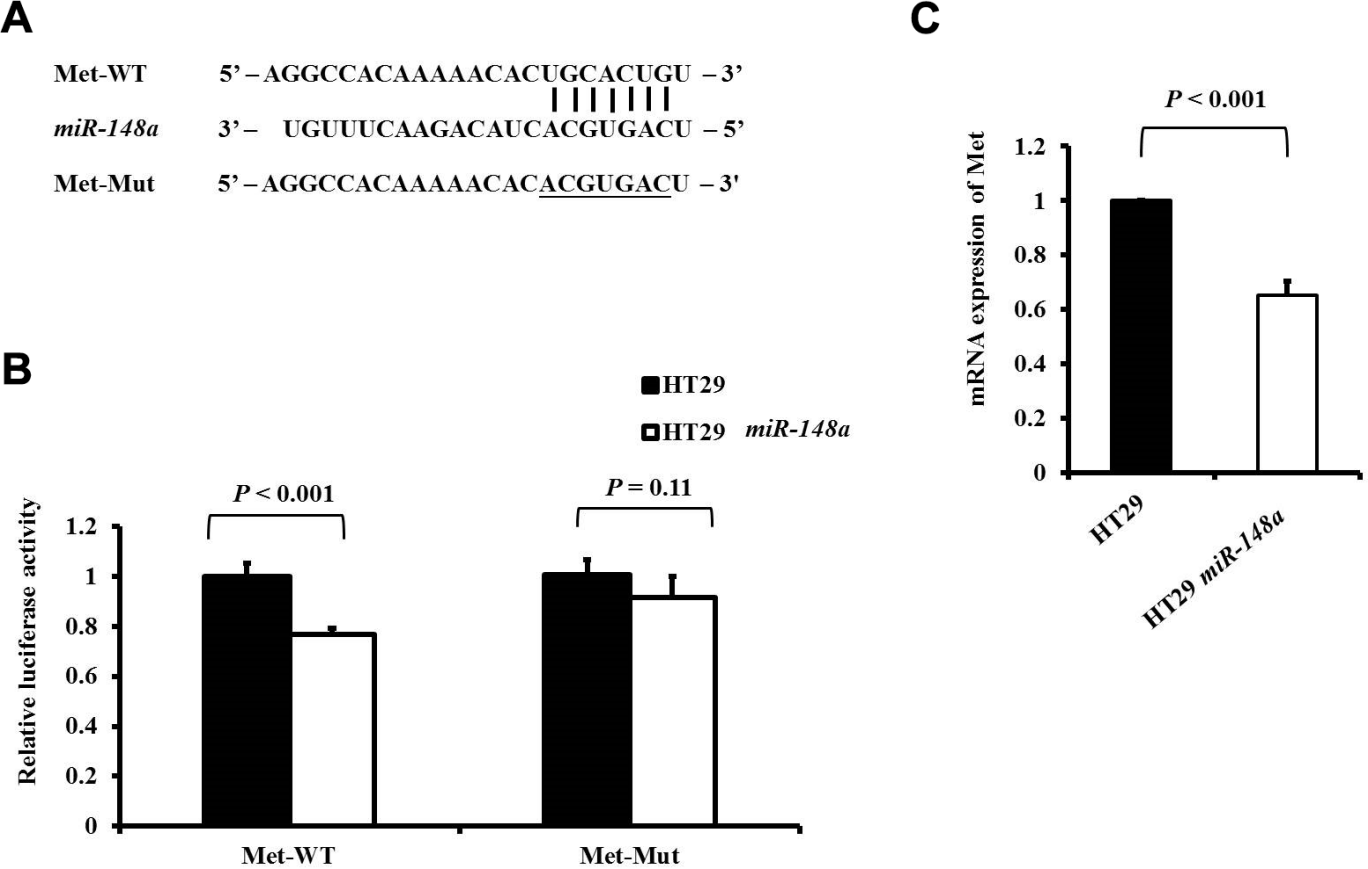
***Met* is a direct target of *miR-148a* in HT-29 cells.** (**A**) *miR-148a* and its putative binding sequences in the 3′-UTR of *Met.* Mutations were generated at the complementary site (underlined) that binds to the seed region of *miR-148a*; (**B**) *miR-148a* overexpression suppressed the activity of firefly luciferase that carried the wild-type (*P* < 0.001) but not mutant 3′-UTR of *Met*; (**C**) The mRNA levels of *Met* were determined using qRT-PCR in stable HT29 and HT29-*miR-148a* cell lines, and the difference was significant in the HT29-*miR-148a* cell line (*P* < 0.001).

*Met*, a tyrosine kinase receptor for hepatocyte growth factor (HGF), plays an important role in *HGF*/*Met*/*Snail* signaling in the EMT and metastasis [27, 28]. The wild-type or mutant 3′-UTR fragments of *Met* were cloned downstream of the firefly luciferase gene to determine whether *Met* is a direct target of *miR-148a* (Figure 2A). It demonstrated by a luciferase reporter assay that the overexpression of *miR-148a* in the HT29 cell line significantly attenuated firefly luciferase activity relative to that in the cell line transfected with the wild-type 3′-UTR of *Met* (*P* < 0.001; Figure 2B), when the predicted 3′-UTR-binding site was mutated and the effect was abolished (*P* = 0.11; Figure 2B). A comparison of the mRNA expression of *c-Met* between the HT29 cells and HT29-*miR-148a* cells revealed that *miR-148a* remarkably downregulated the expression of *c-Met* mRNA (*P* < 0.001; Figure 2C) in the HT29-*miR-148a* cells.

### *miR-148a* directly targets *ROCK1* in the HCT116 cell line to curb the expression of *HIF-1α* and *Mcl-1* under hypoxia

**Figure 3:**
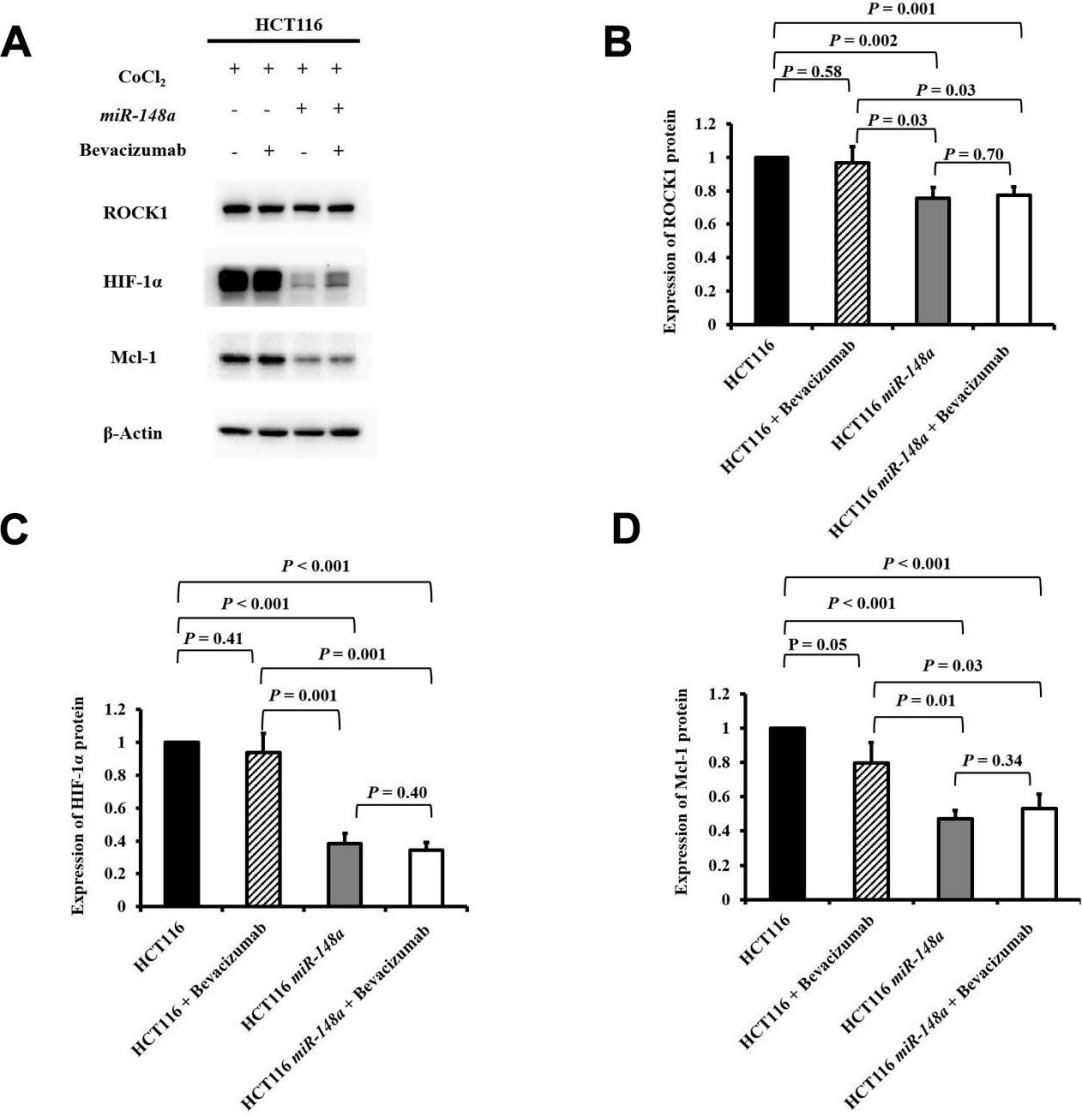
***miR-148a* inhibited the expression of HIF-1α and Mcl-1 proteins by directly targeting *ROCK1* in HCT116 cells under hypoxia.** The protein expression levels of ROCK1, HIF-1α, and Mcl-1 were evaluated under a hypoxic condition generated using CoCl_2_ and in four cell lines (HCT116, HCT116 + bevacizumab, HCT116-*miR-148a*, and HCT116-*miR-148a* + bevacizumab). β-Actin served as an internal control. (**A**) Protein levels of ROCK1, HIF-1α, and Mcl-1; (**B**) The protein expression level of ROCK1 was significantly decreased in the HCT116-*miR-148a* cells but not in the HCT116 cells (*P* = 0.002); (**C**) The protein expression level of HIF-1α was significantly decreased (*P* < 0.001); (**D**) The Mcl-1 protein expression level was markedly decreased (*P* < 0.001).

To examine the functions of *miR-148a* on the activation of *ROCK1*, *HIF-1α*, and *Mcl-1* under hypoxia, we analyzed the protein expression of ROCK1, HIF-1α, and Mcl-1 in the HCT116, HCT116 + bevacizumab, HCT116-*miR-148a*, and HCT116*-miR-148a* + bevacizumab cell lines, separately. The protein expression of ROCK1 was significantly decreased in the HCT116-*miR-148a* cells compared with that in the HCT116 cells (Figure 3A, 3B, *P* = 0.002), but not in the HCT116 + bevacizumab cells (*P* = 0.58, Figure 3A, 3B). Similarly, the protein expression of HIF-1α and Mcl-1 (all *P* < 0.001; Figure 3A, 3C, 3D) was markedly decreased in the HCT116-*miR-148a* cells, but not in the HCT116 + bevacizumab cells (HIF-1α: *P* = 0.41 and Mcl-1: *P* = 0.05; Figure 3A, 3C, 3D).

### *miR-148a directly* targets *Met* in the HT29 cell line to curb the expression of *HIF-1α* and *Mcl-1* under hypoxia

**Figure 4:**
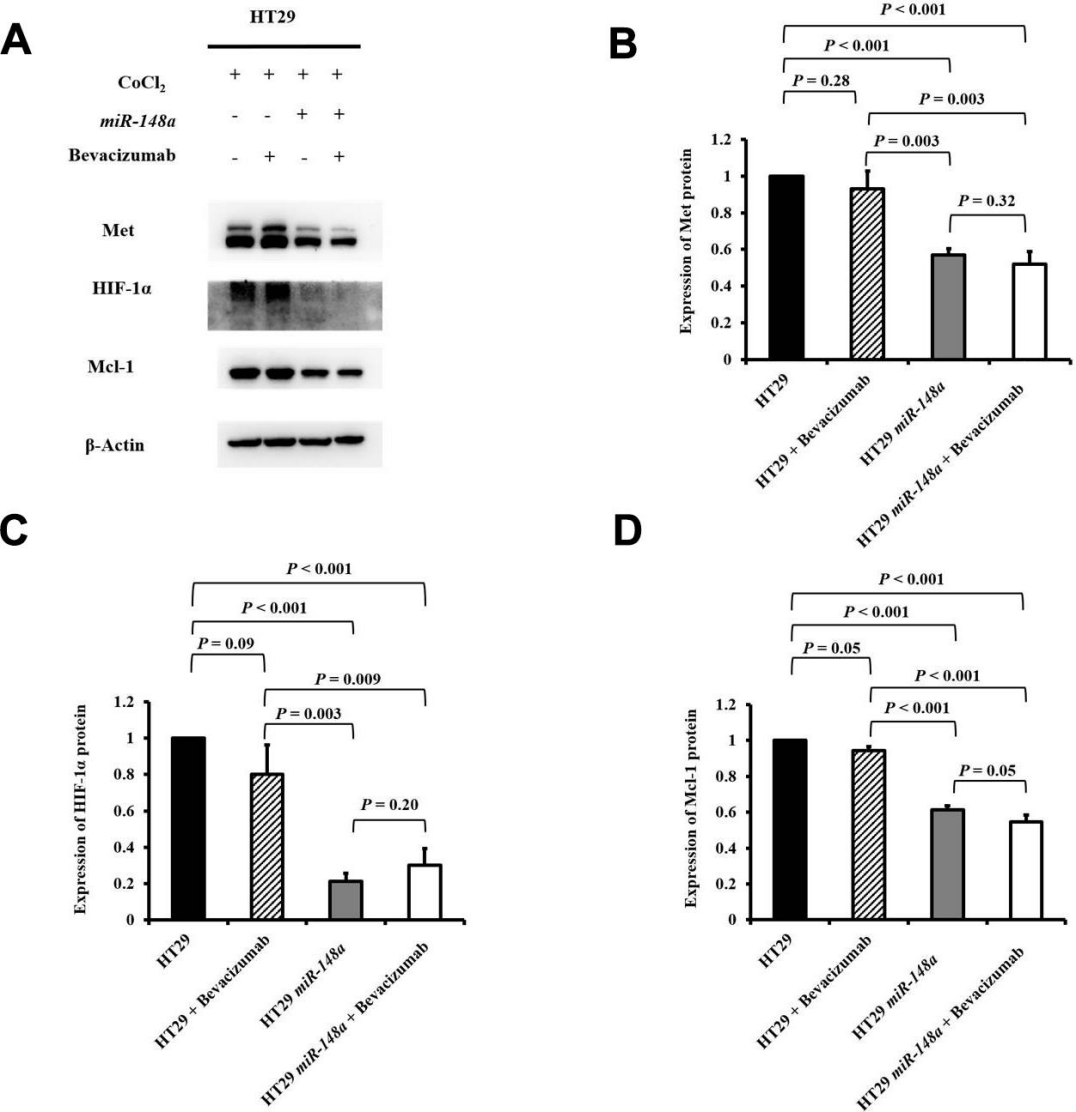
***miR-148a* inhibited the protein expression of HIF-1α and Mcl-1 by directly targeting *Met* in the HT29 cell line under hypoxia.** We created a hypoxic condition by using CoCl_2_ and evaluated the expression levels of HIF-1α and Mcl-1 in four cell lines (HT29, HT29 + bevacizumab, HT29-*miR-148a*, and HT29-*miR-148a* + bevacizumab). β-Actin served as an internal control. (**A**) Protein levels of Met, HIF-1α, and Mcl-1; (**B**) The Met protein expression level was significantly decreased in HT29-*miR-148a* cells but not in HT29 cells (*P* < 0.001); (**C**) The HIF-1α protein expression level was significantly decreased (*P* < 0.001). (**D**) The Mcl-1 protein expression level was markedly decreased (*P* < 0.001).

To research the effect of *miR-148a* on the activation of *Met*, *HIF-1α*, and *Mcl-1* under hypoxia, we further studied the protein expression of Met, HIF-1α, and Mcl-1 in the HT29, HT29 + bevacizumab, HT29-*miR-148a*, and HT29*-miR-148a* + bevacizumab cell lines separately. The protein expression of Met was significantly decreased in the HT29-*miR-148a* cells compared that in with the HT29 cells (Figure 4A, 4B, *P* < 0.001), but not in the HT29 + bevacizumab (*P* = 0.28, Figure 4A, 4B). Similarly, the protein expression of HIF-1α and Mcl-1 (all *P* < 0.001; Figure 4A, 4C, 4D) was significantly decreased in the HT29-*miR-148a* cells, but not in the HCT29 + bevacizumab cells (HIF-1α: *P* = 0.09 and Mcl-1: *P* = 0.05; Figure 4A, 4C, 4D).

### *miR-148a* suppressed *VEGF* secretion and angiogenesis in HCT116 and HT29 colon cancer cells under hypoxia

**Figure 5:**
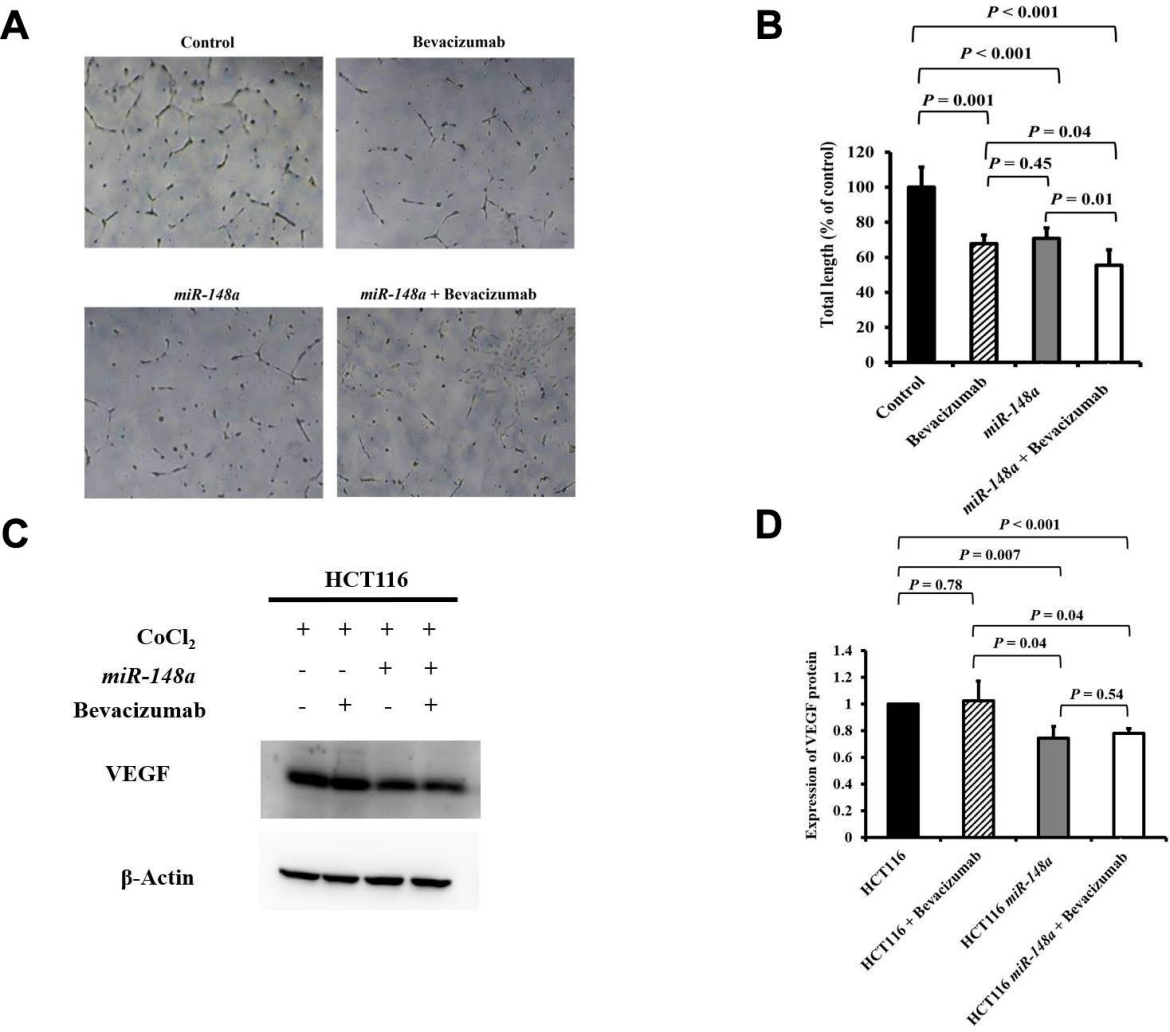
***miR-148a* suppresses VEGF secretion and the angiogenesis of bevacizumab in HCT116 colon cancer cells under hypoxic conditions.** (**A**) Human umbilical vein endothelial cell tube formation assay was performed in four HCT116 cell lines (HCT116, HCT116 + bevacizumab, HCT116-*miR-148a*, and HCT116-*miR-148a* + bevacizumab); (**B**) Both *miR-148a* and bevacizumab significantly inhibited human umbilical vein endothelial cell formation (*P* < 0.001 and *P* = 0.001; respectively); (**C**) VEGF expression levels in HCT116, HCT116 + bevacizumab, HCT116-*miR-148a*, HCT116-*miR-148a* + bevacizumab cells obtained using Western blotting under hypoxia; (**D**) *miR-148a* significantly inhibited VEGF secretion as shown in Western blotting analysis (*P* = 0.007) but not in HCT116 + bevacizumab cells. β-Actin served as an internal control.

**Figure 6:**
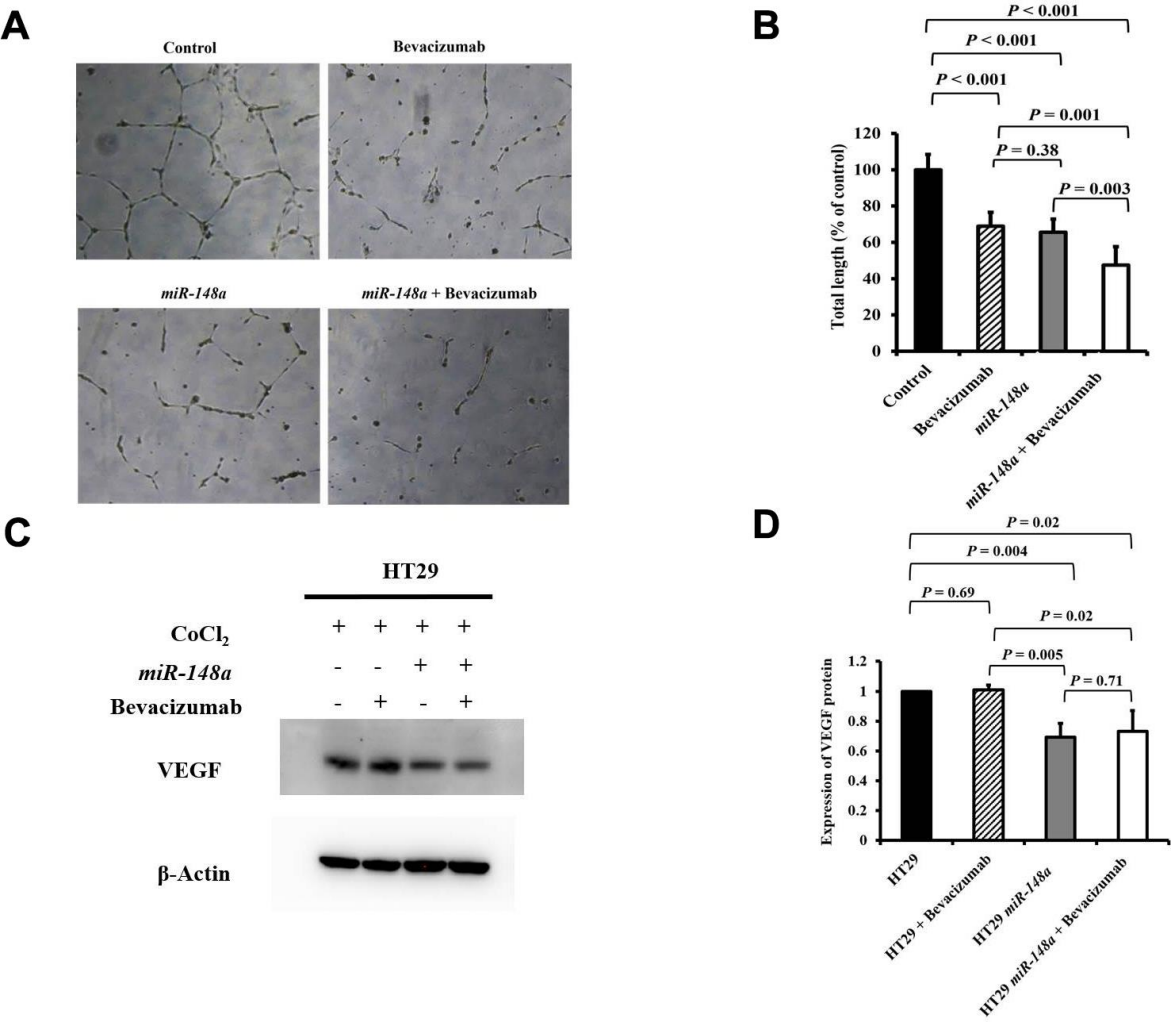
***miR-148a* suppresses VEGF secretion and the angiogenesis of bevacizumab in HT29 colon cancer cells under hypoxic conditions.** (**A**) Human umbilical vein endothelial cell tube formation assay in four HT29 cell lines (HT29, HT29 + bevacizumab, HT29-*miR-148a*, and HT29-*miR-148a* + bevacizumab); (**B**) Both *miR-148a* and bevacizumab significantly inhibited human umbilical vein endothelial cell formation (all *P* < 0.001); (**C**) VEGF expression levels in HT29, HT29 + bevacizumab, HT29-*miR-148a*, and HT29-*miR-148a* + bevacizumab as obtained using Western blotting under hypoxia; (**D**) *miR-148a* significantly inhibited VEGF secretion as shown in the Western blotting analysis (*P* = 0.004) but not in HT29 + bevacizumab cells. β-Actin served as an internal control.

For the nonhypoxic and hypoxic culture condition, we discovered that *miR-148a* uncommonly inhibited the expression of *HIF-1α* and *VEGF* in HCT116 cells (nonhypoxic: *P* = 0.028 and 0.0005; hypoxic: *P* = 0.0007 and 0.02 respectively; Supplementary Figure 2A) and in HT29 cells (nonhypoxic: *P* = 0.0006 and 0.0014; hypoxic: *P* = 0.045 and 0.02, respectively; Supplementary Figure 2B). The antiangiogenesis ability of *miR-148a* was investigated through a tube formation assay in human umbilical vein endothelial cells (HUVECs), and the inhibition of VEGF secretion was assessed through Western blotting. Tube formation was significantly inhibited in the HCT116 cells + bevacizumab group (*P* = 0.001, Figure 5A, 5B), HT29 cells + bevacizumab group (*P* < 0.001, Figure 6A, 6B), HCT116-*miR-148a* cells (*P* < 0.001, Figure 5A, 5B), and HT29-*miR-148a* cells (*P* < 0.001, Figure 6A, 6B). Moreover, *miR-148a* significantly suppressed VEGF secretion in the HCT116-*miR-148a* (*P* = 0.007, Figure 5C, 5D) and HT29-*miR-148a* (*P* = 0.004; Figure 6C, 6D) cells, but bevacizumab did not significantly inhibit VEGF secretion in the HCT116 cells + bevacizumab group (*P* = 0.78, Figure 5C, 5D) or HT29 cells + bevacizumab group (*P* = 0.69, Figure 6C, 6D).

### *miR-148a* overexpression inhibited the viability of HCT116 and HT29 cells

**Figure 7:**
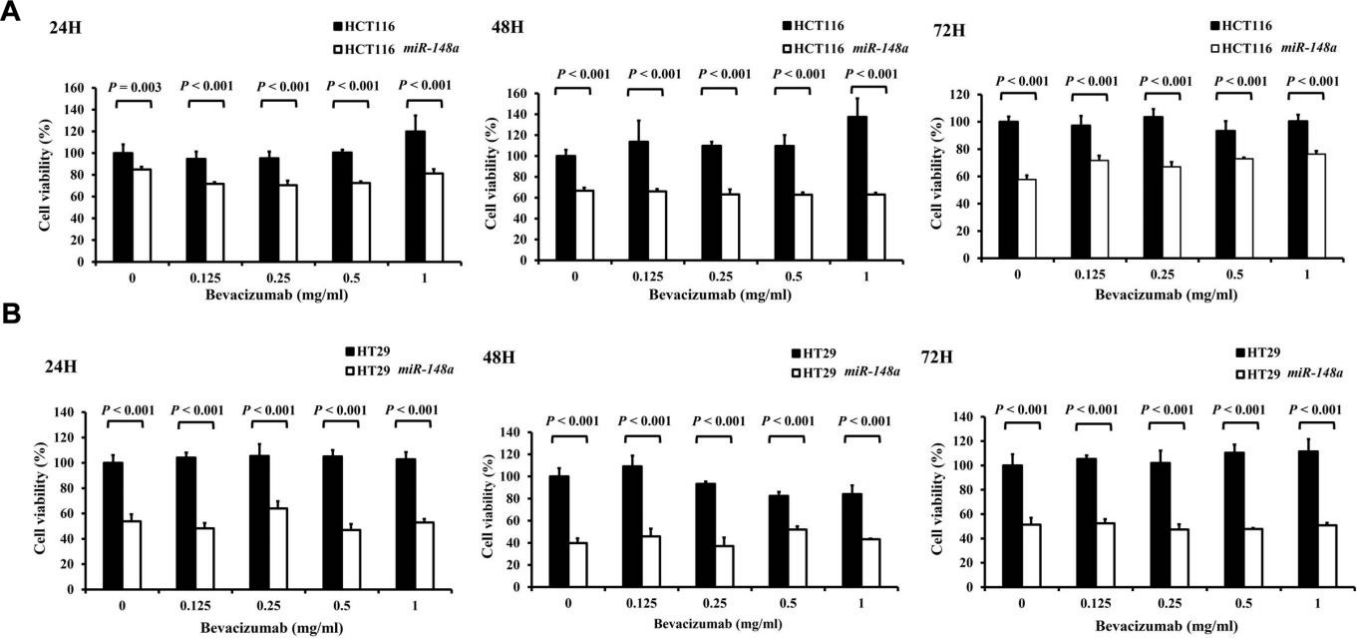
**The anti-cell viability effect of *miR-148a* on HCT116 and HT-29 colon cancer cells.** Four cell lines (HCT116, HCT116-*miR148a*, HT29, and HT29-*miR-148a*) were treated with different concentrations of bevacizumab for 24, 48, and 72 h. (**A**) HCT116 and HCT116-*miR148a* cells; (**B**) HT29 and HT29-*miR-148a* cells. *miR-148a* significantly inhibited cell viability regardless of the concentration of bevacizumab at 24, 48, and 72 h (all *P* < 0.001).

*miR-148a* remarkably inhibited cell viability regardless of the concentrations of bevacizumab at 24, 48, and 72 h (all *P* < 0.001; Figure 7). This result indicated the *miR-148a* alone could directly suppress the proliferation of colon cancer cells.

### Synergistic antitumorigenic effect of *miR-148a* and bevacizumab on nude mice

**Figure 8:**
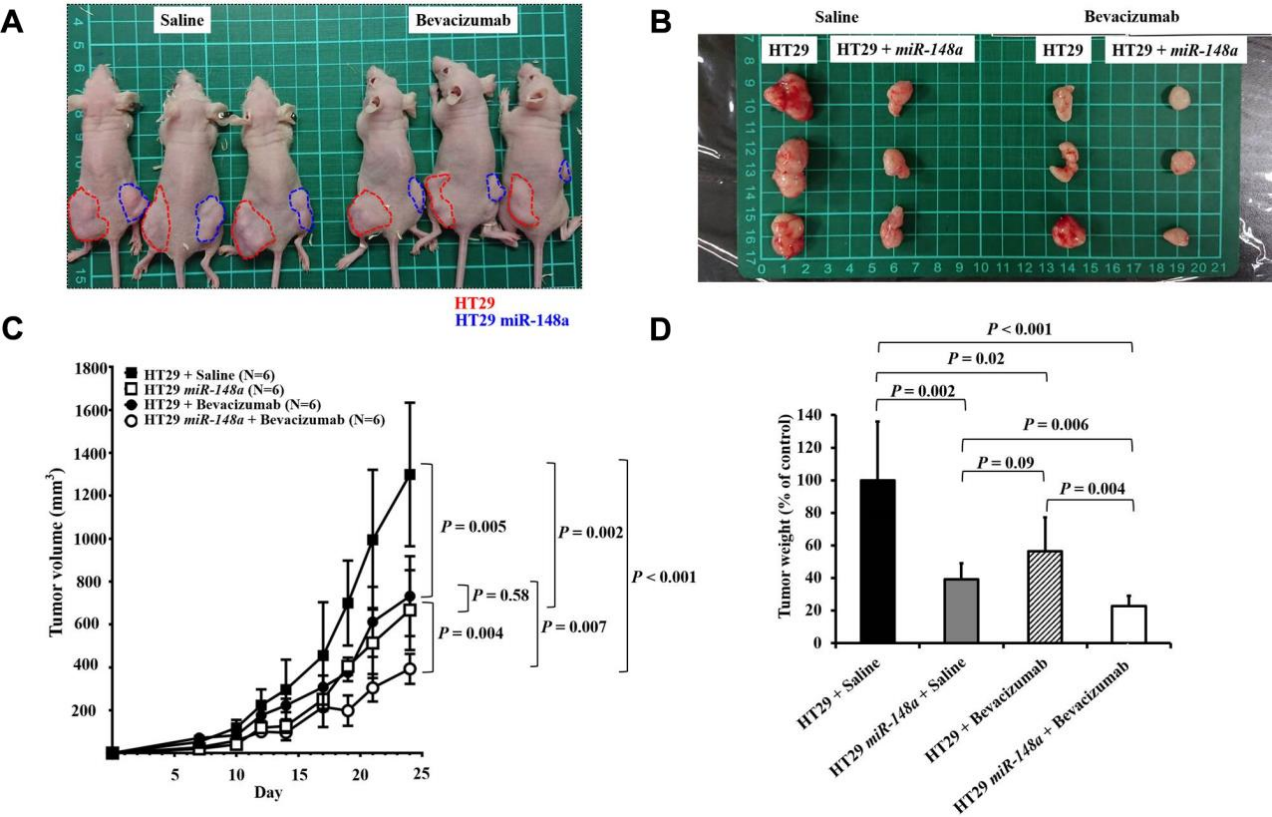
**Synergistic anti-tumorigenic effect in animal study.** To validate the role of *miR-148a* in tumorigenesis and evaluate the effect of *miR-148a* overexpression on tumor growth *in vivo*, *miR-148a overexpression* and NC clones with scrambled pCDH-NC were injected subcutaneously in 8-week-old nude mice to allow tumor growth (red circle: NC; blue circle: *miR-148a overexpression*). (**A**) One week after implantation, the mice were assigned to two groups and saline (Left) or bevacizumab (Right) was injected at the tumor site to evaluate the synergistic anti-tumorigenic effect. (**B**) Mice that received bevacizumab and *miR-148a overexpression* had significantly smaller cancer lumps than those that received saline and NC. (**C**) After the tumor-bearing mice were sacrificed at 3 weeks after tumor cell seeding, tumor burdens were analyzed. Mice that received bevacizumab had significantly smaller cancer lumps than those that received saline (*P* = 0.005) but larger lumps than those that received bevacizumab + *miR-148a overexpression* (*P* = 0.007). (**D**) Mice that received bevacizumab had significantly lower tumor weight than those that received saline (*P* = 0.02) but higher tumor weight than those that received bevacizumab + *miR-148a overexpression* (*P* = 0.004).

One week after implantation, mice were assigned to two groups, and saline (Left; Figure 8A) or bevacizumab (Right; Figure 8A) was injected at the tumor site to evaluate the synergistic antitumorigenic effect. Mice that received bevacizumab had significantly smaller cancer lumps (*P* = 0.005; Figure 8B, 8C) and lower tumor weight (*P* = 0.02; Figure 8D) than did those that received saline. Furthermore, the coadministration of *miR-148a* and bevacizumab exerted significant synergistic effects on the reduction of tumor volume (*P* = 0.007, Figure 8C) and tumor weight (*P* = 0.004, Figure 8D). The results suggest that similar to the effect of bevacizumab, *miR-148a* overexpression leads to a prominent reduction in tumor cell proliferation in animal models.

### Association between serum expression of *miR-148a* and therapeutic response in mCRC patients

**Figure 9:**
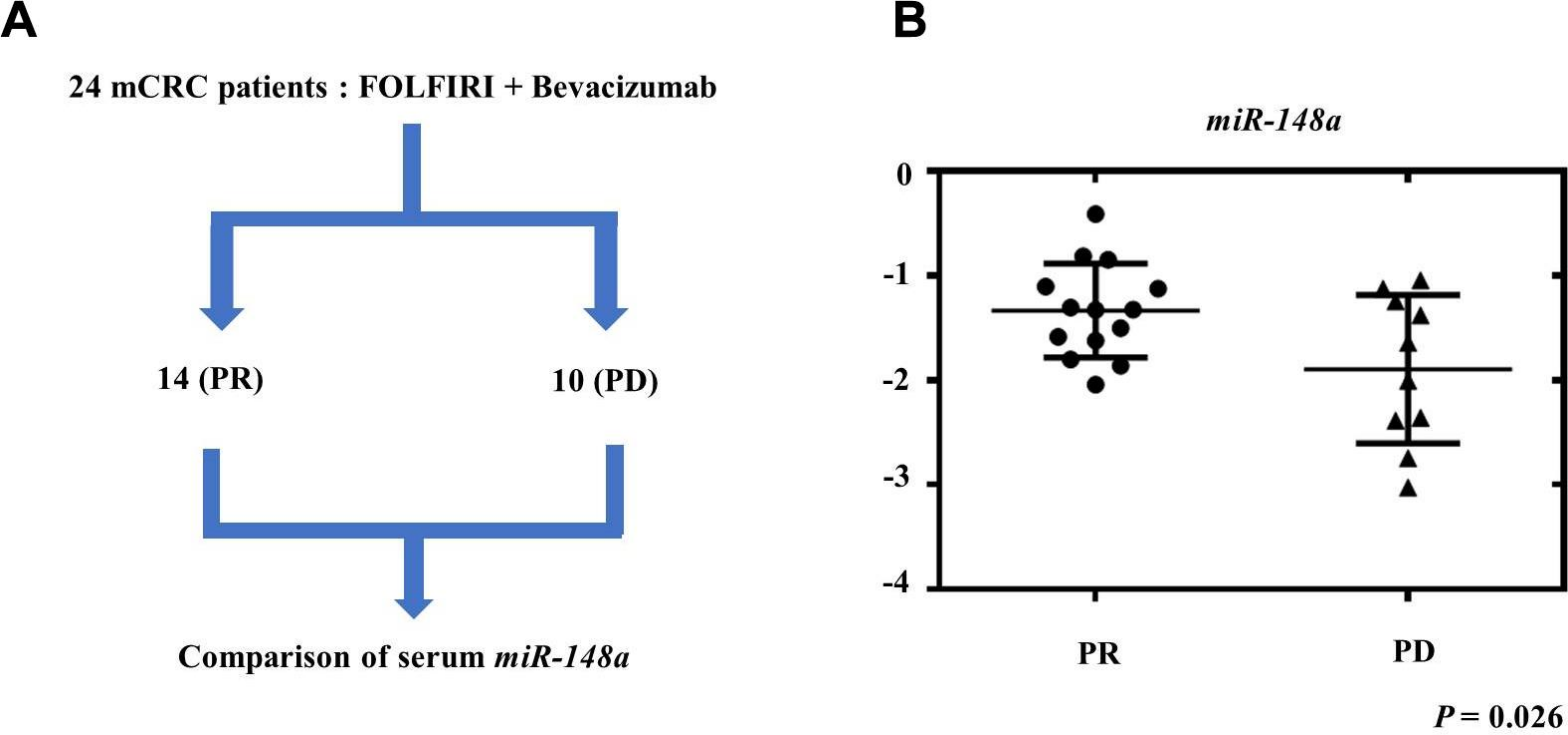
**Relationship between therapeutic response and serum *miR-148a* expression in mCRC patients.** We collected the serum samples of 24 mCRC patients before they received treatment with bevacizumab plus FOLFIRI as first-line regimen. (**A**) After treatment, 14 patients showed partial response (PR) and 10 patients had progressive disease (PD). (**B**) *miR-148a* expression was significantly higher in the serum samples of the 14 PR patients than in those of the 10 PD patients (*P* = 0.026).

To evaluate the role of *miR-148a* expression in mCRC patients treated with bevacizumab, we collected the serum samples of 24 mCRC patients before they received bevacizumab plus FOLFIRI as the first-line treatment. Among these patients, 14 showed a partial response (PR), and 10 had progressive disease (PD) (Figure 9A). The patients who exhibited PR had significantly higher serum *miR-148a* expression than those who had PD (*P* = 0.026, Figure 9B). This result indicated that mCRC patients who exhibited *miR-148a* overexpression receiving bevacizumab plus FOLFIRI had a more favorable treatment response than those with lower *miR-148a* expression.

## DISCUSSION

One of the main findings of the current study is that *miR-148a* could decrease angiogenesis in and increase the apoptosis of colon cancer cells via direct downregulation of *ROCK1* and *c-Met* and their relevant pathways. Even under hypoxic conditions, *miR-148a* efficiently inhibited the expression of *HIF-1α*, *VEGF*, and *Mcl-1*. We re-verified that *miR-148a* can inhibit angiogenesis in and decrease the viability of CRC cells whether it is *in vitro* or *in vivo* conditions. Moreover, a notable synergistic effect of *miR-148a* and bevacizumab on antitumorigenesis was observed in the animal model.

ROCK1 is an essential effector kinase of Rho GTPases and plays a vital role in regulating tumor invasion and metastasis [25, 26]. In 2011, Zheng et al. demonstrated that *miR-148a* suppressed tumor cell invasion and metastasis by downregulating *ROCK1* in gastric cancer cells [29]. *c-Met*, a tyrosine receptor, plays a key role in the EMT and metastasis in hepatocellular carcinoma [27, 28]. In 2014, Zhang et al. reported that *miR-148a* may inhibit *Met/Snail* signaling and may negatively regulate the EMT and metastasis of hepatoma cells [17]. We confirmed that *miR-148a* directly targeted the 3′-UTR of *ROCK1* and *c-Met* in colon cancer cell lines in the current study.

Angiogenesis is a complicated process in which the formation of new blood vessels are from an endothelial precursor in malignancies [6]. This process is tightly regulated and mediated by a group of ligands [30, 31]. Both HIF-1α and VEGF play critical roles in angiogenesis and tumor progression [32, 33]. The process of programmed cell death and a homeostatic mechanism that helps maintain cell populations during normal cell development and aging is called apoptosis. It maintains balance between the generation of new cells and the loss of cells in normal tissues. Impaired apoptosis is an important step in tumorigenesis. Apoptosis has two underlying signaling pathways: extrinsic and intrinsic [34, 35]. *miR-148a* deactivates the intrinsic mitochondrial pathway via *Bcl-2* inhibition and tumor apoptosis induction in CRC [8, 16]. Mcl-1, a prosurvival protein of the *Bcl-2* family, exhibits anti-apoptotic ability. It is the main regulator of cell death [36] and targets the phosphorylation of the protein kinase Raf-1 to inactivate cell death signaling pathways [37, 38].

Many studies have revealed the role of *miR-148a* in multiple malignancies, including in inhibiting the growth of pancreatic and prostate cancer cells [39, 40] and suppressing angiogenesis in breast cancer [41]. We previously demonstrated that *miR-148a* suppressed VEGF through the downregulation of the *pERK/HIF-1α/VEGF* pathway, thereby inhibiting angiogenesis [7]. The overexpression of *miR-148a* assisted apoptosis and curbed proliferation by directly targeting *c-Met in vitro* and improved the tumor response to irradiation *in vivo* [19]. Nersisyan et al. reported that the *ITGA5* gene and *PRNP* gene were upregulated in Caco-2 cells exposed to chemical hypoxia. The Cancer Genome Atlas Colon Adenocarcinoma cohort reported that elevated expression levels of both these genes were associated with poor prognosis in patients with CRC. Further, *miR-148a* may directly interact with both genes to influence tumor progression and metastasis [42]. Thus, *miR-148a* functions as a potential tumor suppressor through diverse mechanisms. In this study, we affirmed that *miR-148a* indirectly inhibited the expression of HIF-1α and Mcl-1 by directly binding to *ROCK1* and *c-Met*, thereby enhancing apoptosis of colon cancer cells and decreasing angiogenesis in tissues containing such cells. Notably, we also found that bevacizumab acts as an angiogenesis inhibitor by inhibiting VEGF-A and arresting tumor growth. However, it could not inhibit VEGF secretion. Nevertheless, whether bevacizumab leads to tumor cell apoptosis through the downregulation of Mcl-1 secretion remains uncertain. The overexpression of *miR-148a* synergistically enhanced the antitumorigenic and apoptotic effects of bevacizumab, thereby yielding better therapeutic outcomes than bevacizumab-only treatment *in vitro* and *in vivo*.

The study has some limitations. First, the sample size was relatively small. Future studies should enroll a larger cohort to confirm the predictive value of *miR-148a* in patients with mCRC. Second, the synergistic effects of *miR-148a* and bevacizumab on tumor volume and weight reduction could be demonstrated in the animal model, but not in colon cancer cell lines. Third, the evidence regarding ROCK1/c-Met, HIF-1α/VEGF, and Mcl-1 in this study is based on the results of other studies. This finding should be validated in future investigations.

Finally, our research demonstrated that *miR-148a* downregulated *HIF-1α/VEGF* and *Mcl-1* by directly targeting *ROCK1/c-Met* to decrease angiogenesis and increase the apoptosis of colon cancer cells. Clinically, we demonstrated that patients with mCRC with serum *miR-148a* overexpression have more a favorable therapeutic response than those undergoing the combination therapy of chemotherapy and bevacizumab (standard dose; 5 mg/kg). Therefore, the *miR-148a* status possesses prognostic/predictive value in patients with advanced CRC treated with anti-VEGF biological agents and has clinical implications in improving therapeutic strategies and designing personalized treatment for this malignancy.

## MATERIALS AND METHODS

### Study design

**Figure 10:**
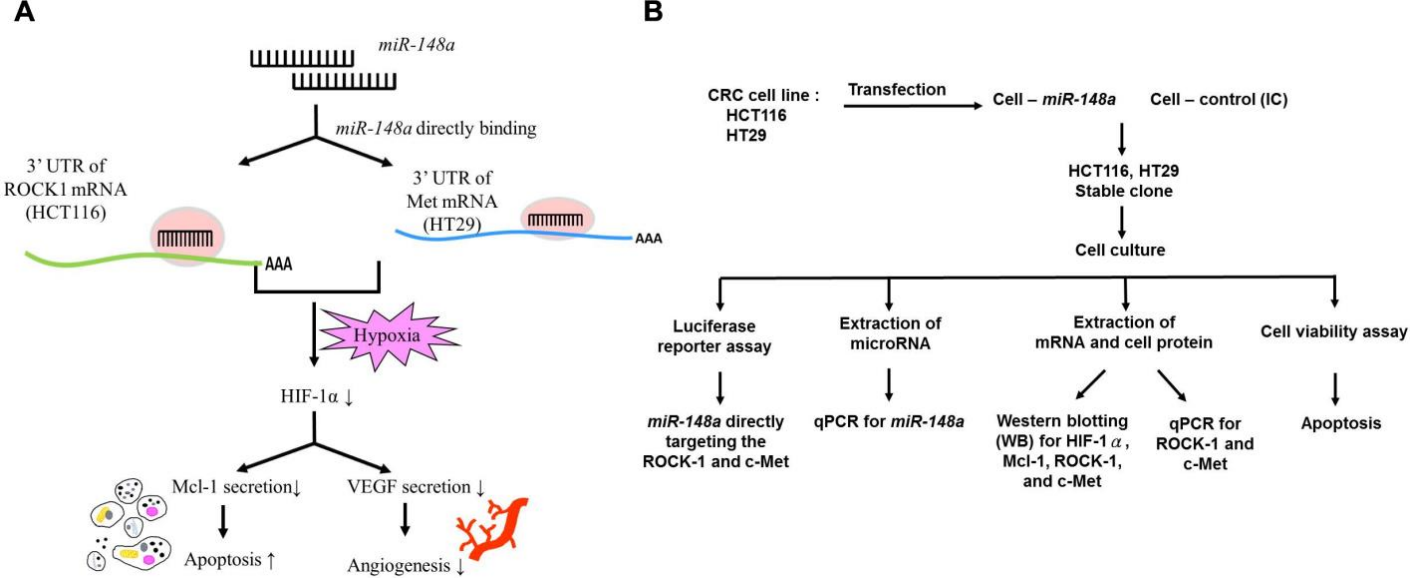
**Study hypothesis and design.** (**A**) We hypothesized that *miR-148a* inhibits the secretion of VEGF and Mcl-1 through the inactivation of *HIF-1α* by directly targeting *ROCK1* and *c-Met*. This induced the apoptosis of and reduced angiogenesis in cancer cells. (**B**) *In vitro*, we transfected *miR-148a* into HCT116 and HT29 cells and established stable CRC clones. The luciferase reporter assay was performed to prove the direct targeting of *ROCK1* and *c-Met* by *miR-148a*. The protein levels of HIF-1α, Mcl-1, ROCK1, and Met were examined through Western blotting, and the mRNA levels of *ROCK1* and *c-Met* were tested through RT-PCR. The cell viability assay was used to examine the apoptosis.

We demonstrated the relationship between *miR-148a* and VEGF indirect downregulation by targeting *HIF-1α* under nonhypoxic and hypoxic conditions in our previous study [7]. Herein, the TargetScan program (https://targetscan.org) and the isomiRTar portal (https://isomirtar.hse.ru/) were made use of identifying potential genes which *miR-148a* directly targets. We only considered the conserved genes which carried conserved sequences. We used Gene Ontology (http://geneontology.org) software to discover the function of *miR-148a* target genes. A previous bioinformatics analysis of pathways revealed that *miR-148a* directly targets *ROCK1* and *c-Met*, thereby affecting the function of Mcl-1 [43]. Accordingly, we hypothesized that *miR-148a* inhibits the secretion of VEGF and Mcl-1 proteins by directly targeting *ROCK1* and *c-Met* and can therefore enhance the apoptosis of and downregulate angiogenesis in cancer cells (Figure 10A). The design of the cell-line study is presented in Figure 10B, and the process of the animal study is detailed in Supplementary Figure 3.

### Cell lines and cell line authentication

Five colon cancer cell lines—HCT116, HT29, SW480, SW620, and Caco-2—were used for the transfection and proliferation of *miR-148a*; the HCT116 and HT29 cells exhibited higher fold changes and relative proliferation after transfection than the other cell lines (Supplementary Figure 4A, 4B). In addition, the activation of the *RAS-RAF* pathway was related to increase VEGF-induced angiogenesis. We also evaluated the role of *miR-148a* in and the effects of *BRAF* mutations (HT29 cells) and *KRAS* mutations (HCT116 cells) on angiogenesis. We purchased the HCT116 and HT29 cells from the Bioresource Collection and Research Center (Hsinchu, Taiwan) and American Type Culture Collection (Manassas, VA, USA), respectively. We cultured the all of cell lines in Dulbecco's modified Eagle’s medium (Gibco, Grand Island, NY, USA) supplemented with 10% fetal bovine serum (Gibco), 100 IU/mL penicillin (Gibco), and 100 μg/mL streptomycin (Gibco) in a humid atmosphere containing 5% CO_2_ at 37° C. HUVECs, a part of the Angiogenesis Starter Kit (Thermo Fisher Scientific, Inc., Waltham, MA, USA), was also assayed.

We resuscitated the HCT116 and HT29 cell lines and cultured them for 2 weeks after delivery from the provider and were then transfected with the pCDH vector (System Biosciences, Palo Alto, CA, USA) expressing *miR-148a*. We used the transfected cell lines in subsequent experiments. Through transduction in the target cells, the pCDH expression vector could integrate into genomic DNA, thus providing stable, long-term expression of the target gene. The genome of the transfected cells was different from that of the wild-type cells because of the genomic integration of the expression vector. Therefore, the pCDH-transfected cells were no longer identified by their original genomic status compared with the wild-type cells in the cell line authentication test. Because the genomic changes were expected, the cell lines used in this study were not subjected to an authentication test.

### Patient tissue samples

To assess the correlation between the effect of bevacizumab (Avastin, Roche, Basel, Switzerland) treatment and *miR-148a* expression levels, patients with mCRC who received FOLFIRI plus bevacizumab (Avastin) were classified into two groups according to their treatment response: The PR cohort, which comprised 14 patients, and the PD cohort, which comprised 10 patients. The serum samples of the patients in the two cohorts were collected for RNA extraction. All clinical samples were collected after written informed consent was obtained from each participant. The study protocol was approved by the Institutional Review Board of Kaohsiung Medical University Hospital (KMUHIRB-G(II)20190039).

### Establishment of vectors with overexpressed *miR-148a*

To evaluate the functional effects of overexpressed *miR-148a*, the pCDH vector (System Bioscience, Mountain View, CA, USA) was used as the overexpressed *miR-148a* system. We constructed the pCDH-*miR-148a* plasmid by intercalating the *miR-148a* PCR product into multiple cloning sites of the pCDH vector. The following PCR primer sequences were used for *miR-148a* cloning: GCCTGAATTCATGCTTTTAACGAGTTATTCTTC and CTAGGCGGCCGCGCCTTGCCCCTCCCCCAAGGA. The forward and reverse primers were extended by inclusion of GAATTC and GCGGCCGC sequences, respectively, creating *EcoR1* and *Not1* restriction sites at their 5′ end, respectively. The *miR-148a* overexpression vectors were confirmed through direct DNA sequencing.

### Establishing stable clones

The HCT116 and HT29 cells (5 × 10^5^) were seeded and transfected them with the negative-scrambled pCDH vector or the pCDH–*miR-148a* plasmid (400 ng) by using Lipofectamine 3000 (Thermo Fisher Scientific). The transfected cells were cultured in standard culture media supplemented with 12 μg/mL puromycin (Thermo Fisher Scientific) over 4 weeks to achieve pCDH-negative or pCDH–*miR-148a* stable clone selection. The TaqMan miRNA quantitative PCR (RT-qPCR) assay (Applied Biosystems, Waltham, MA, USA) was conducted to confirm the stable expression of the transfected plasmid.

### Luciferase reporter assay

To verify *miR-148a*-directed binding on targeted mRNA, two 3′-UTR segments including one wild-type and the other mutant type were cloned and integrated into the pMirTarget Vector (OriGene Technologies, Inc., Rockville, MD, USA), respectively. Both the wild-type and mutant 3′-UTR segments of *c-Met* or *ROCK1* were constructed, because *c-Met* and *ROCK1* were predicted to be the potential target genes of *miR-148a*. The cells (1 × 10^4^) were seeded in a 96-well plate for 24 h and co-transfected with two plasmids—wild-type or mutant 3′-UTR construct and pTK-Green Renilla Luc Vector (Thermo Fisher Scientific)—by using Lipofectamine 3000 (Thermo Fisher Scientific). After transfection for 48 h, a luciferase reporter assay was performed using the Pierce™ Renilla-Firefly Luciferase Dual Assay Kit (Thermo Fisher Scientific) according to the manufacturer’s instructions. For the normalization of firefly activity, the Renilla luciferase activity was used as the internal control.

### RNA extraction and cDNA preparation

Approximately 10^7^ cells were prepared for the extraction of RNA including mRNA and miR. Total RNA purification was performed using Qiagen RNAeasy Columns (Qiagen, Germantown, MD, USA) according to the manufacturer’s instructions. To synthesize the cDNA of *miR-148a* or U6 for the detection of *miR-148a* expression levels, 100 ng of total RNA with the unique primer (Applied Biosystems) was used, and to synthesize the cDNA of mRNA for studying the mRNA expression level of *miR-148a*-mediated genes, 2 μg of total RNA with random hexamer primers (Applied Biosystems) was applied.

### *miR-148a* expression levels in patient samples and CRC cell lines

The TaqMan miR RT-qPCR assay (Applied Biosystems) was applied to quantify the *miR-148a* expression level. RT-qPCR was performed using the Applied Biosystems 7900HT Real-Time PCR System. The relative *miR-148a* expression level in each serum sample was normalized to the internal control of U6 snRNA in accordance with the following equation: log10(2^−ΔCt^), where ΔCt = (Ct_miR-148a_ − Ct_U6_).

### mRNA expression level

The mRNA expression levels of *miR-148a*-mediated genes were quantified with SYBR Green (Applied Biosystems) by using the Applied Biosystems 7900HT Real-Time PCR System. The relative expression level in each sample was normalized to the internal control of glyceraldehyde 3-phosphate dehydrogenase and was evaluated using the following formula: (2^−ΔΔCt^).

### HUVEC tube formation assay

Angiogenesis was induced *in vitro* by using the Angiogenesis Starter Kit (Gibco, Grand Island, NY, USA) according to the manufacturer’s manual. In brief, a day before the tube formation assay was performed, the Geltrex Matrix was thawed at 4° C and then bottom coated in a 24-well plate at 37° C for 30 min. Subsequently, the HUVECs were seeded on the Geltrex Matrix–coated plate and incubated for 24 h, after which the conditional medium collected from transfected cells was added to each well. Following 24 h of incubation, cell images were taken using the Edipse Ti-U inverted microscope system (Nikon, Inc., Melville, NY, USA), and the total length of the tube formed was measured using ImageJ software (National Institutes of Health, Bethesda, MD, USA).

### Cell viability assay

To study the anticancer effects of *miR-148a*, we first treated the HCT116, HCT116-*miR-148a*, HT29, and HT29-*miR-148a* cells with various concentrations (0 mg/ml, 0.125 mg/ml, 0.25 mg/ml, 0.5 mg/ml, and 1 mg/ml) of bevacizumab for 24, 48, and 72 h, respectively, and evaluated cell viability by using the 3-(4,5-dimethylthiazol-2-yl)-2,5-diphenyltetrazolium bromide (MTT) assay kit (Sigma-Aldrich, St. Louis, MO, USA). We seeded the cells (5 × 10^3^ cells/well) into a 96-well culture plate and incubated the plate overnight at 37° C before treatment with the appropriate dose of bevacizumab (Avastin) (0, 0.125, 0.25, 0.5, or 1 mg/mL) (Roche). After 24-, 48-, and 72-h incubation, 20 μL of 5 mg/mL MTT was added to each well and incubated at 37° C for 2 h. The medium was replaced with 100 μL of dimethyl sulfoxide to dissolve the precipitate. Thereafter, absorbance was measured at 570 nm on a 96-well microplate reader (BioTeK Instruments, Winooski, VT, USA).

### Western blotting and antibodies

All the cells were harvested and lysed with ice-cold RIPA buffer (Merck Millipore, Burlington, MA, USA), protease inhibitor cocktail (Sigma-Aldrich), and phosphatase inhibitor cocktail (Sigma-Aldrich). Equal amounts (30 μg) of protein were resolved through sodium dodecyl sulfate-polyacrylamide gel electrophoresis and were transferred onto polyvinylidene difluoride (PVDF) membranes (Merck Millipore). After blocking with 5% skim milk for 1 h, the PVDF membranes were incubated with primary antibodies, such as anti-VEGF 165A (Abcam PLC, Cambridge, England, UK), anti-ROCK1 (Abcam PLC, Cambridge, England, UK), anti-Met (Cell Signaling Technology, Danvers, MA, USA), anti-HIF-1α (Cell Signaling Technology), anti-Mcl-1 (Cell Signaling Technology), and anti-β-Actin (Sigma-Aldrich), overnight at 4° C. Following washing with tris-buffered saline containing Tween 20 (TBS-Tween 20), the PVDF membranes were incubated with the secondary antibody at room temperature for 1 h. After the membranes were washed, immunoreactive proteins were detected using a SuperSignal™ West Femto Maximum Sensitivity Substrate (Thermo Fisher Scientific).

### Inhibition of *HIF-1α* and *VEGF* and *Mcl-1* expression by *miR-148a* under hypoxia

Seo et al. [44] demonstrated CoCl_2_ to be a hypoxia mimetic agent. Therefore, we used CoCl_2_ to create a hypoxic culture condition and demonstrated the ability of *miR-148a* to inhibit *HIF-1α*, *VEGF*, and *Mcl-1* expression under such a condition.

### Xenograft study

We further validated the role of *miR-148a* in anti-tumorigenesis and evaluated the effect of *miR-148a* overexpression on tumor growth *in vivo*. Six-week-old male Balb/c nude mice were purchased from BioLASCO Taiwan, Co., Ltd. (Taipei, Taiwan) and were maintained in the Center for Laboratory Animals of Kaohsiung Medical University. At 8 weeks of age, the mice were subcutaneously injected with *miR-148a* overexpression and NC clones with scrambled pCDH-NC for tumor growth (red circle: NC; blue circle: *miR-148a* overexpression). Each mouse received two subcutaneous injections of 1 × 10^7^ HT29 cells (either pCDH-negative or pCDH–*miR-148a*) into the bilateral flank for the implantation of two tumors. One week after implantation, the mice were assigned into two groups—saline only or bevacizumab. The mice received an intraperitoneal injection of bevacizumab (2.5 mg/kg) (Roche) or an equal volume of saline twice per week. The tumor diameter was measured, and tumor volume was calculated using the following formula: V = (length × width^2^)/2. After the tumor-bearing mice were sacrificed at 3 weeks after tumor cell seeding, tumor burdens were analyzed. All animal experiments were performed in an Association for Assessment and Accreditation of Laboratory Animal Care International-accredited facility. The animal handling procedures were in accordance with the protocols approved by the Institutional Animal Care and Use Committee of Kaohsiung Medical University (IACUC Approval No: 108165).

### Statistical analysis

All statistical analyses were performed using SPSS V21.0 (IBM SPSS, Chicago, IL, USA). Data are expressed as the mean ± standard deviation values of at least three independent experiments. Significant differences between two groups were determined using Student’s *t*-test analyses. A *P* value of <0.05 was considered statistically significant.

## Supplementary Material

Supplementary Figures
