# Supplementary material for: MicroRNA-148a induces apoptosis and prevents angiogenesis with bevacizumab in colon cancer through direct inhibition of ROCK1/c-Met via HIF-1α under hypoxia
Source: Aging (Albany NY). 2022 Aug 22;14(16):6668–88. doi: 10.18632/aging.204243 (PMC9467409; doi:10.18632/aging.204243)
Supplement: Supplementary Figures [file aging-14-204243-s001.pdf]

SUPPLEMENTARY FIGURES

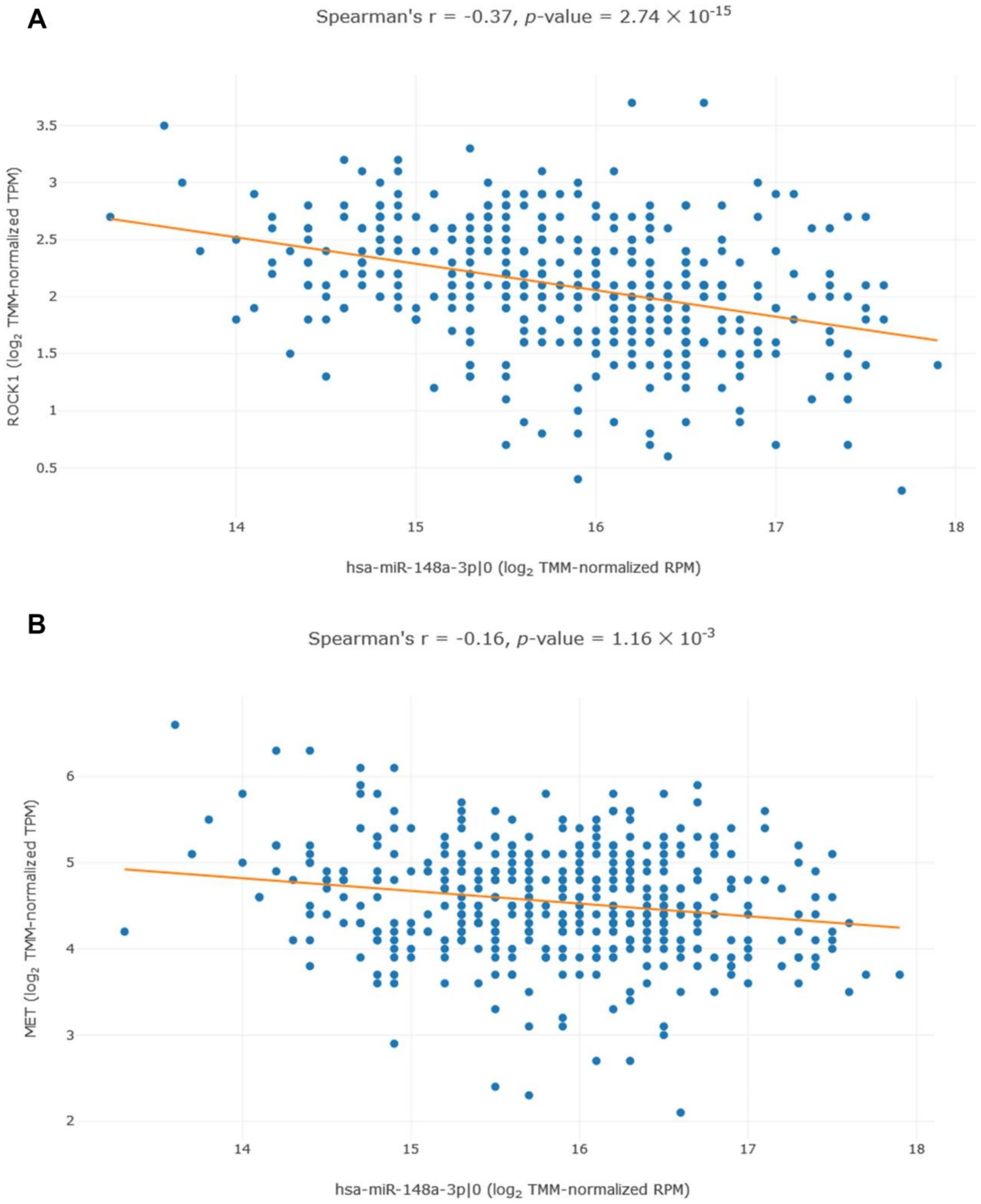

Supplementary Figure 1. Two candidate genes, *ROCK1* and *c-Met*, were selected from the isomiRTar portal. (**A**) *miR-148a* is significantly anti-correlated with *ROCK1* (**B**) *miR-148a* slightly anti-correlated with *c-Met*.

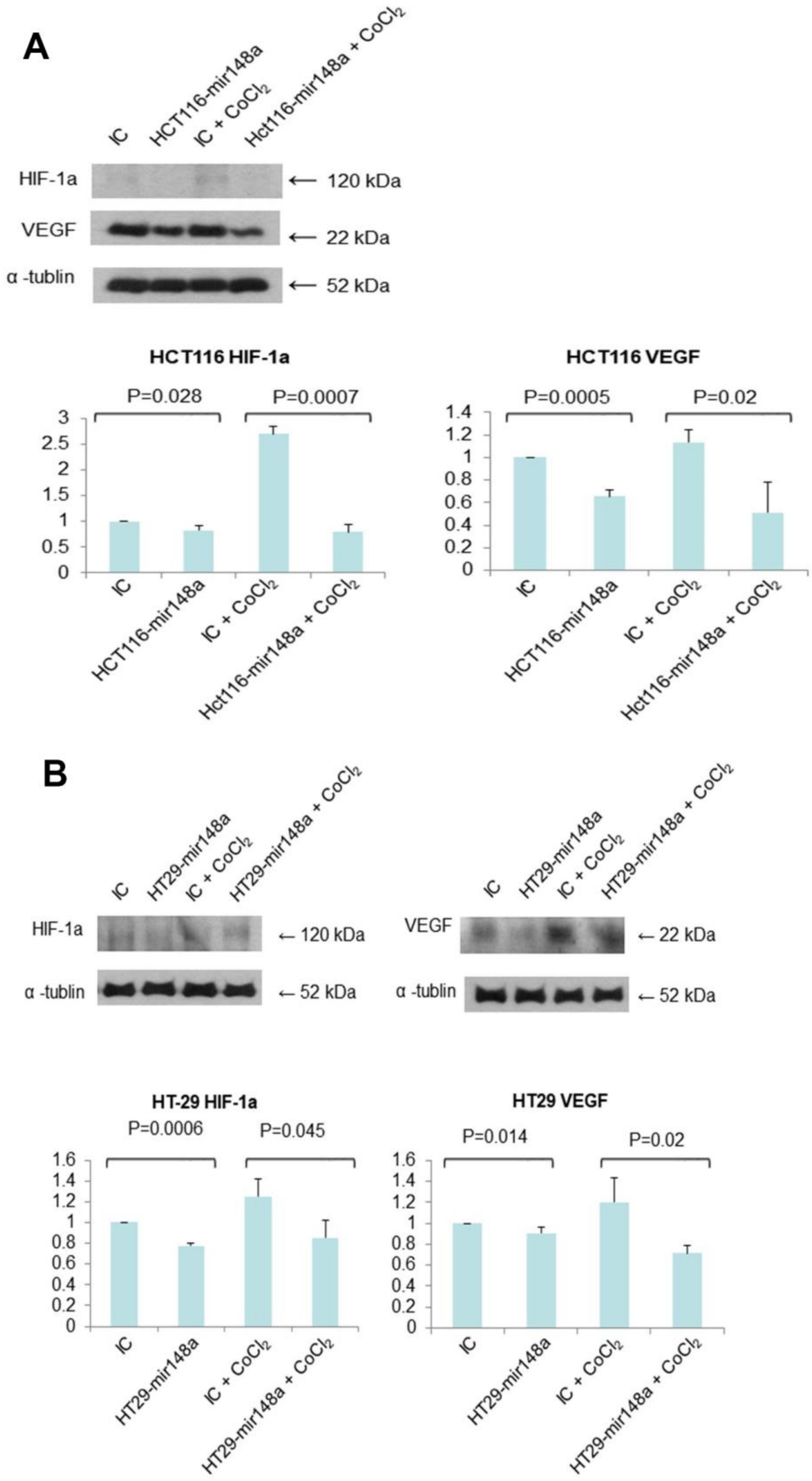

Supplementary Figure 2. *miR-148a* suppressed the secretions of VEGF and HIF-1α under hypoxic condition (created by CoCl2). (**A**) *miR-148a* could significantly inhibit the expressions of *HIF-1α* and *VEGF* in HCT116 (non-hypoxic: P = 0.028 and 0.0005; hypoxic: P = 0.0007 and 0.02, respectively). (**B**) *miR-148a* could significantly inhibit the expressions of *HIF-1α* and *VEGF* in HT29 (non-hypoxic: P = 0.0006 and 0.0014; hypoxic: P = 0.045 and & 0.02, respectively).

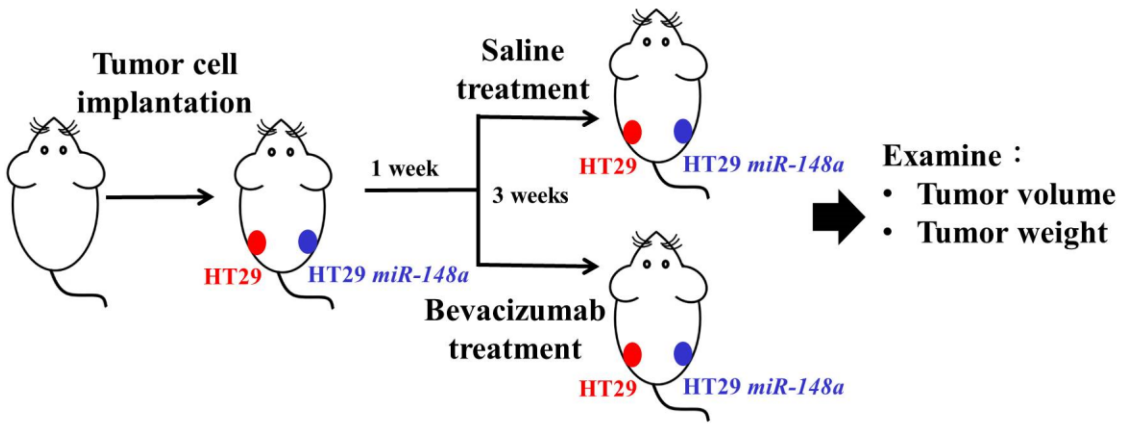

Supplementary Figure 3. The process of the animal study. At 8 weeks of age, the mice subcutaneously injected with *miR-148a overexpression* and NC clones (HT29 cells) with scrambled pCDH-NC for tumor growth (red circle: NC; blue circle: *miR-148a overexpression*). One week after implantation, the mice were assigned into two groups—saline only or bevacizumab. The mice received an intraperitoneal injection of bevacizumab (2.5 mg/kg) or an equal volume of saline twice per week. After the tumor-bearing mice were sacrificed at 3 weeks after tumor cell seeding, tumor burdens were analyzed.

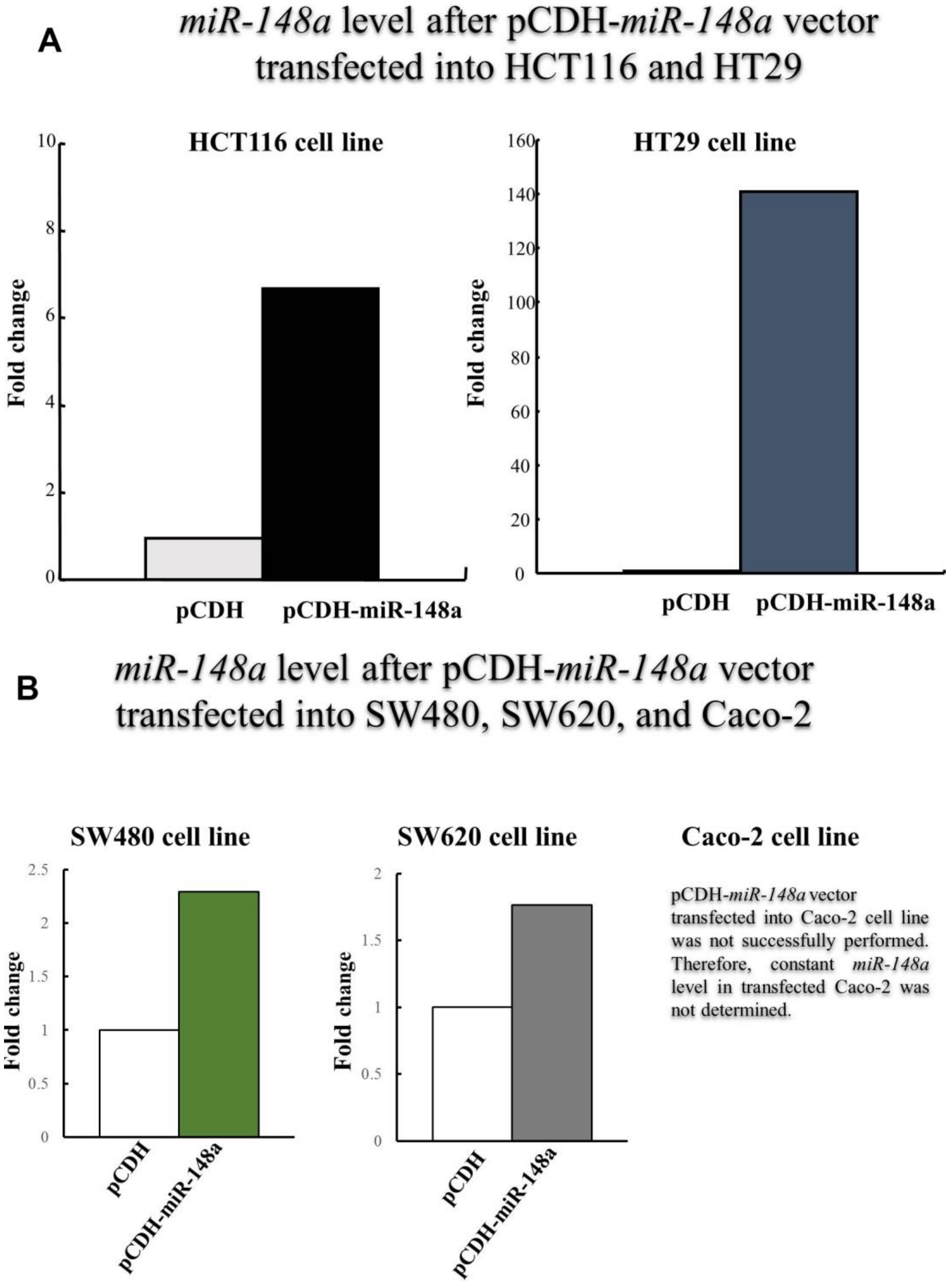

Supplementary Figure 4. *miR-148a* was transfected into five colon cancer cell lines. (**A**) *miR-148a* was successfully transfected into HCT116 (7-fold) and HT29 cells (140-fold). (**B**) Transfection of *miR-148a* into SW480 cells (2.3-fold) and SW620 cells (1.75-fold) was not significant, and transfection was not successful in Caco-2 cells.
